# Metformin and Prostate Cancer: a New Role for an Old Drug

**DOI:** 10.1007/s11934-017-0693-8

**Published:** 2017-04-25

**Authors:** Jessica Whitburn, Claire M. Edwards, Prasanna Sooriakumaran

**Affiliations:** 1 Nuffield Department of Surgical Sciences, University of Oxford, Botnar Research Centre Old Road, Oxford, OX3 7LD UK; 2 Department of Urology, University College London Hospitals NHS Foundation Trust, London, UK

**Keywords:** Metformin, Biguanide, Cancer, Prostate, AMPK

## Abstract

**Purpose of Review:**

Since epidemiological studies first demonstrated a potential positive effect of metformin in reducing cancer incidence and mortality, there has been an increased interest in not only better understanding metformin’s mechanisms of action but also in exploring its potential anti-cancer effects. In this review, we aim to summarise the current evidence exploring a role for metformin in prostate cancer therapy.

**Recent Findings:**

Preclinical studies have demonstrated a number of antineoplastic biological effects via a range of molecular mechanisms. Data from retrospective epidemiological studies in prostate cancer has been mixed; however, there are several clinical trials currently underway evaluating metformin’s role as an anti-cancer agent. Early studies have shown benefits of metformin to inhibit cancer cell proliferation and improve metabolic syndrome in prostate cancer patients receiving androgen deprivation therapy (ADT).

**Summary:**

While the body of evidence to support a role for metformin in prostate cancer therapy is rapidly growing, there is still insufficient data from randomised trials, which are currently still ongoing. However, evidence so far suggests metformin could be a useful adjuvant agent, particularly in patients on ADT.

## Introduction

Prostate cancer (PCa) remains the most commonly diagnosed male cancer in the Western world and is the second leading cause of cancer-related deaths in men [1]. Over the last 10 years, increasing evidence is demonstrating that biguanide drugs such as metformin may be beneficial in the prevention and treatment of a range of cancers, including prostate cancer [2]. Metformin is the most commonly used oral anti-diabetic drug in the world. It is also used in the treatment of polycystic ovary syndrome, and is being investigated as a potential antiviral agent. It has been in clinical use for more than 50 years and has a good safety record with limited toxicity. Lower cancer incidence and cancer-specific deaths have been reported among diabetics on metformin compared to diabetics on other anti-diabetic medications [3•, 4]. These results are in keeping with in vitro work, which has shown metformin to be anti-proliferative to a range of cancer cell lines, and in vivo work where metformin has been shown to inhibit the growth of cancer xenografts [5, 6]. Higher insulin and c-peptide levels have been associated with poorer outcomes in cancer patients [7, 8], and metformin has been shown to be effective at reducing insulin levels, even in non-diabetic patients [9]. Given its excellent safety profile, low cost, and minimal side effects, it is an attractive candidate as a potential cancer therapeutic. However, we still have limited knowledge of the specific molecular mechanisms underlying its beneficial properties.

## Metformin: Mechanism of Action

**Fig. 1:**
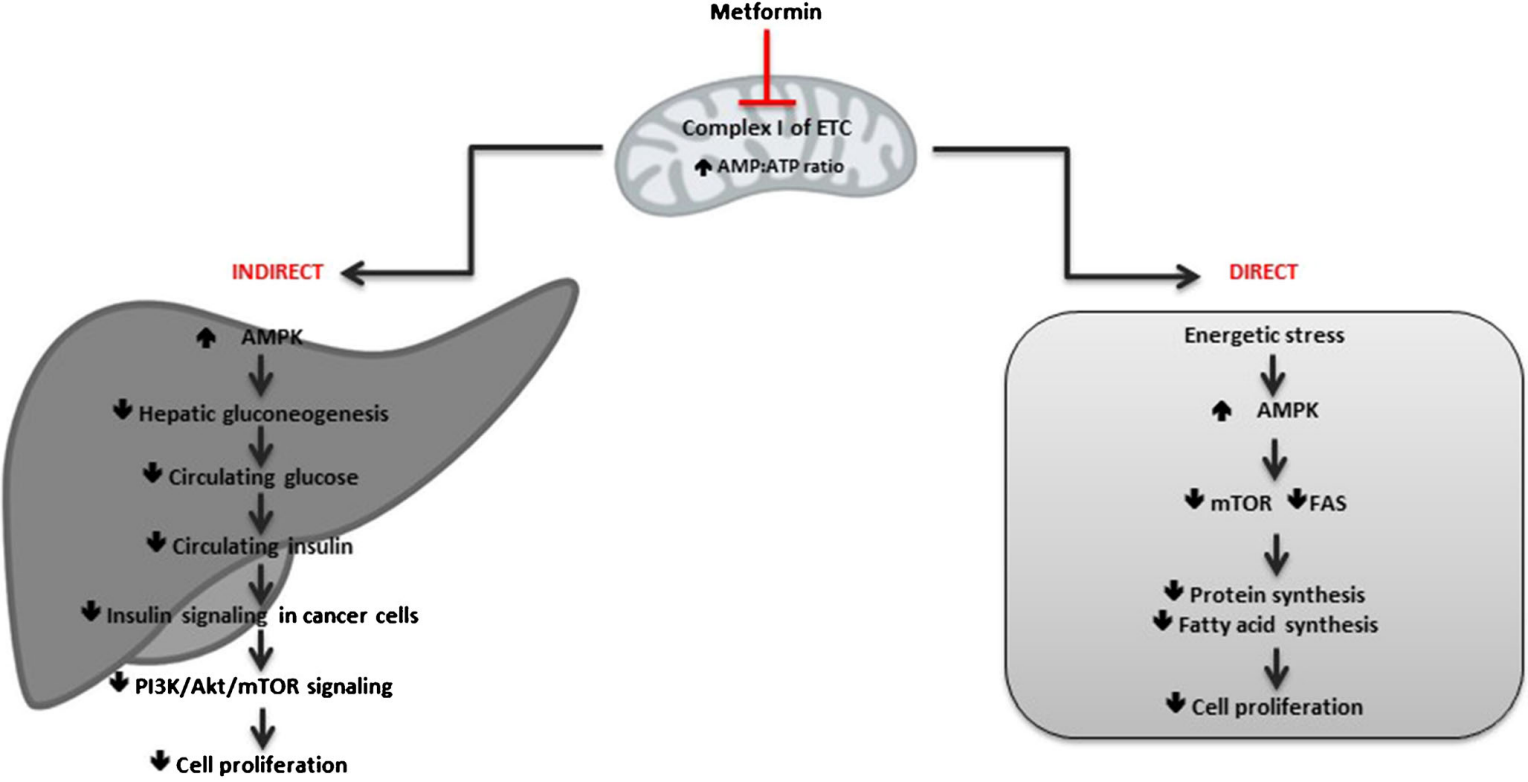
Overview of the direct and indirect anti-cancer effect of metformin

Metformin was originally derived from *Galega officinalis* (French lilac), a plant reputedly used to treat diabetes-like conditions in medieval Europe [10]. Despite its widespread use in the treatment of type 2 diabetes, details of the mechanism of action of metformin in this disease were only recently elucidated, and gaps in knowledge remain [11]. It is thought to exert its anti-cancer effect via two mechanisms—*directly* by acting on the tumour and *indirectly* by lowering systemic insulin levels (Fig. 1). The *direct* (insulin-independent) effect is via inhibition of the mitochondrial electron transport chain (ETC) and consequent activation of the enzyme adenosine mono-phosphate-activated protein kinase (AMPK) [12]. AMPK is an energy sensing/signalling protein that is a central control point for maintaining energy homeostasis. It detects an increase in the ratio of AMP to ATP secondary to cellular stresses (e.g. glucose deprivation, hypoxia, and oxidative stress). When activated, it increases cellular ATP by inhibiting anabolic pathways and stimulating catabolic pathways to produce ATP [13, 14]. Activation of AMPK leads to downstream inhibition of mammalian target of rapamycin complex-1 (mTORC1) signalling, and activation of the tumour suppressor tuberous sclerosis complex 2 (TSC2, tuberin) [15]. mTOR is a key mediator of the phosphatidylinositol-3-kinase/protein kinase B/Akt (PI3K/PKB/Akt) signalling pathway, which is one of the most frequently deregulated pathways in human cancer [16]. Inhibition of mTOR leads to attenuation of protein synthesis and tumour cell growth and proliferation through downstream targets, 4E-BP1 and p70S6K1 [17–20]. Additionally, inhibition of the Krebs cycle may have further direct effects on other metabolic pathways such as lipid synthesis and beta-oxidation that are known to be important in prostate cancer metabolism [21]. Fatty acid synthase (FAS), a key enzyme of lipogenesis, is known to be upregulated in cancer to allow high rates of de novo fatty acid production. AICAR, an activator of AMPK, has been shown to inhibit FAS in prostate cancer cells [22] which may further contribute to metabolic stress.

The indirect (insulin-dependent) actions of metformin occur via inhibition of hepatic gluconeogenesis. Activation of AMPK in the liver leads to inhibition of the transcription of key gluconeogenesis genes, and stimulates glucose uptake in muscle, thus reducing fasting blood glucose and insulin levels [10, 23]. Insulin is known to have mitogenic and pro-survival effects, with tumour cells often expressing high levels of the insulin receptor. High insulin levels are known to be an adverse prognostic factor for a number of cancers, including breast, colon, and prostate cancer [8, 24, 25], and metformin has been shown to be able to lower systemic insulin levels, even in non-diabetic patients [9]. Reduced circulating insulin leads to a subsequent down-regulation of the phosphoinositide-3-kinase (PI3K) axis. The PI3K pathway is involved in growth, proliferation, differentiation, and motility, and following the androgen receptor (AR) pathway is the second major driver of prostate cancer growth. The indirect actions are systemic and therefore do not require metformin to be able to accumulate within the tumour which maybe important for poorly vascularised tumours.

Which mechanism predominates in prostate cancer is unknown but it is likely that both pathways provide anti-cancer benefits. Also, it still remains unclear whether AMPK activation is essential for metformin activity, as its ability to inhibit mTORC1 has also been demonstrated via AMPK-independent pathways [26]. Further research is ongoing to better clarify the mechanisms that predominate in the cancer setting.

## Epidemiological Evidence

Metformin’s inhibitory effects on various pro-oncogenic pathways prompted epidemiologist to retrospectively analyse the effect of metformin treatment on type 2 diabetic patients with cancer. Evans and colleagues were the first to show that taking metformin may be associated with reduced risk of cancer in patients with type 2 diabetes, and that the longer the period of exposure the greater the benefit [3•]. A more recent study in the same geographical setting showed that in a cohort of 8000 patients with type 2 diabetes, cancer was diagnosed in 7.3% of metformin users compared with 11.6% of non-users, and that higher doses of metformin were associated with the greatest reduction in cancer risk [27]. Further studies have compared metformin therapy to other anti-diabetic therapies and found that while diabetics had an increased cancer mortality compared to non-diabetics, those on metformin had a lower cancer mortality compared to those taking other anti-diabetic medications such as sulfonylureas or insulin [4, 28]. The effects of metformin have also been analysed in organ-specific disease. In prostate cancer, several studies have found a beneficial effect of metformin in reducing prostate cancer incidence and improving overall survival [29–33]. A recent large population-based study found that metformin users were approximately 16% less likely to be diagnosed with prostate cancer than non-users, with an inverse relationship between prostate cancer risk and duration, intensity of use, and cumulative dose [34•]. Despite the size of these epidemiological studies, they are all retrospective examining only diabetic patients, and there is considerable confounding and heterogeneity between the studies. To date, only three randomised trials examining metformin’s effect on cancer survival have been published, all in the late disease setting. These have found no difference in overall survival in patients treated with metformin in combination with standard systemic therapies [35–37]. However, a recent study by Higurashi et al. [38] examined metformin’s role in chemoprevention of colorectal cancer and found that low-dose metformin for 1 year reduced the prevalence and number of metachronous colorectal adenomas or polyps in patients with high risk of adenoma recurrence after polypectomy, with no serious adverse events during the trial. In view of this, many questions still remain with regards to metformin’s role in oncology therapy. Will metformin be an effective anti-cancer agent in non-diabetic patients? Is the anti-diabetic dose the best anti-cancer dose? Will metformin only be effective in certain subgroups of patients, or in certain malignancies? Will metformin’s effects be limited to cancer prevention or does it have a role in cancer treatment? There are currently several randomised clinical trials being undertaken to answer these questions both in prostate cancer and other malignancies, and the results of these are eagerly awaited.

## Role in Preventing Metabolic Syndrome

Alongside its potential anti-cancer role, clinical trials have also examined a role for metformin in preventing androgen deprivation therapy (ADT)-induced metabolic syndrome in prostate cancer. Whilst ADT is the mainstay of treatment for men with advanced prostate cancer, improving patient’s survival, it can also cause significant morbidity and a decline in quality of life (QOL). Body composition changes, insulin resistance, sexual dysfunction, fatigue, reduced bone mineral density (BMD), hyperlipidaemia, metabolic syndrome, and acute coronary syndrome are all reported adverse effects of ADT as a consequence of reduced levels of circulating testosterone and raised insulin levels [39, 40]. Men with prostate cancer have higher rates of non-cancer mortality and cardiovascular morbidity, and some of this excess risk can be attributed to androgen deprivation therapy [41]. Studies have shown that even short-term use of ADT significantly increases fat mass and decreases insulin sensitivity in men with prostate cancer [42]. Metabolic syndrome is not only associated with cardiovascular disease (CVD) but may also have an adverse effect on prostate cancer prognosis. Insulin has been shown to promote local androgen synthesis by prostate cancer cells, which is thought to represent a resistance mechanism to castration [43]. A retrospective study showed the presence of metabolic syndrome was associated with a shorter median time to progression and shorter median overall survival in prostate cancer patients receiving ADT [44]. If metformin is able to lower the hyperinsulinaemia and improve the metabolic syndrome seen, then there will be a strong rationale to examine the benefits of combining metformin with ADT, with a potential role for metformin in improving both the tolerability and efficacy of ADT. There is currently only one randomised trial reporting on this which found that a combination of metformin with a low glycaemic index-diet and exercise programme improved the abdominal girth, weight, BMI, and systolic blood pressure of men with prostate cancer beginning ADT; however, no difference was noted in the biochemical markers of insulin resistance [45••]. Further trials are needed to determine if metformin has a role in reducing ADT-induced metabolic syndrome, and if this corresponds in a reduction in CVD-related deaths, prostate cancer-specific death, and all-cause mortality.

## Biological Effects

Metformin has been shown to inhibit the proliferation of a range cancers, including breast, endometrial, ovarian, colon, and prostate [5, 18, 46–50]. Its effect can be seen in both in vitro cell lines and in mouse tumour models. Interestingly, Ben Sahara et al. [5] found that while metformin was anti-proliferative to prostate cancer cells, normal epithelial prostate cells were barely affected by metformin suggesting a cancer-specific effect. However, it is important to note that many of these experiments have used exceedingly high doses, up to 100 to 300 times the conventional anti-diabetic dose. More recently, there have been several ‘window-of-opportunity’ biomarker trials of metformin in cancer patient’s pre-surgery examining immunohistochemical markers, typically using anti-diabetic therapeutic doses. Metformin has been shown to reduce expression of Ki67, a marker of cell proliferation in breast, endometrial, and prostate cancers [51–53, 54••, 55]. Joshua et al. matched biopsy and prostatectomy samples in 24 non-diabetic patients given a short (median duration 41 days) course of metformin prior to prostatectomy. They found metformin was well tolerated and significantly reduced the Ki67 index by almost 30% per patient. There was also a non-significant trend towards PSA reduction in these patients. Weaknesses of this study include the fact that it was a small pilot trial (*n* = 22), not placebo controlled, and that the tissue examined was obtained by different methods (biopsy vs prostatectomy); however, it provides strong rationale for further studies to not only examine disease and survival end-points but to also incorporate tissue and serum markers to better elucidate metformin’s actions.

Interestingly, metformin’s biological effects are not limited to inhibiting cancer proliferation, but include effects on epithelial to mesenchymal transition (EMT), bone turnover, the androgen receptor, and cancer stem cells. EMT is an important step in prostate cancer invasion and metastases, conferring upon cancer cells the ability to invade basement membranes and metastasize to distant sites. EMT can be characterised by expression of certain markers by the cell. Epithelial markers include E-cadherin, cytokeratin, and desmoplakin, while mesenchymal markers include vimentin, slug, snail, and twist. Expressions of twist and vimentin have been shown to be predictive of biochemical recurrence in prostate cancer patients after radical prostatectomy for localised disease [56]. In melanoma and breast cancer cells, metformin is able to inhibit expression of markers of EMT via activating AMPK [57, 58]. It has also been shown to reverse the mesenchymal phenotype of prostate cancer cells in vitro and inhibit their invasive phenotype [59].

Prostate cancer most commonly metastasises to the bone leading to skeletal-related events. In combination with this, ADT leads to loss of bone mineral density and increased risk of fracture. Metformin has been shown to enhance osteoblast and inhibit osteoclast differentiation in vitro [60, 61], and to prevent bone loss in ovariectomized rats [62], raising the possibility that metformin may also be protective against cancer-induced bone disease in these high-risk patients.

The androgen receptor (AR) plays a vital role in prostate cancer, regulating a multitude of events including proliferation, apoptosis, migration, invasion, and differentiation. ADT remains the standard treatment for advanced prostate cancer; however, almost all patients treated with ADT will eventually relapse into castration-resistant prostate cancer (CRPC). Studies have demonstrated that persistent AR signalling remains the key driver in the progression to CRPC, and AR down-regulation is considered a preventive strategy for prostate cancer [63]. Metformin has been shown to reduce AR protein levels in a dose-dependent manner in androgen receptor-positive cell lines [49], and repress the AR signalling pathway via down-regulation of AR mRNA [64], again supporting a potential role for metformin in combination with ADT therapy.

Cancer stem cells (CSCs) are a subset of tumour cells that are resistant to many anti-cancer treatments, and are able to self-renew and regenerate the various cell types that make up a tumour [65]. Prostate CSCs are known to be resistant to most conventional cancer therapies and are thought to contribute to local invasion and bone metastasis [66] as well as the development of CRPC [67]. Metformin has been shown to effectively target breast and pancreatic CSCs, and it has also been shown to enhance the effectiveness of standard therapies [68, 69].

Iliopoulos et al. found metformin in combination with the chemotherapeutic drug doxorubicin was more effective than either drug alone at blocking breast, prostate, and lung cancer growth and preventing relapse in mouse xenograft models. Interestingly, metformin had a comparable effect on tumour regression and preventing relapse when combined with a fourfold decreased dose of doxorubicin that was not effective as a monotherapy, suggesting a complementary role for metformin, potentially allowing lower doses of toxic drugs to be used, improving their tolerability and side effects [70]. Indeed, there have been a number of studies showing synergistic or enhanced effects of metformin when used in combination with a range of other therapies including commonly used medications such as statins and aspirin [71–73]. The combination of metformin with ADT has been shown to enhance the reduction of prostate tumour growth in mouse models [74], and metformin has been shown to increase the sensitivity of prostate cancer cells to radiotherapy treatment [75, 76]. This provides a strong rationale for the addition of metformin to current clinical trial treatment regimens, and indeed, metformin has been added as an arm to the STAMPEDE trial (Systematic Therapy for Advancing or Metastatic PCa), a UK trial which has recruited greater than 4000 patients to date.

## Conclusions

Metformin is an attractive anti-cancer agent in view of its well-documented safety record, well-characterised pharmacodynamics profile, and low cost as a generic drug. Over the last 10 years, interest in this field has grown exponentially with a combination of epidemiological, clinical, and preclinical data supporting a potential anti-cancer role for metformin. However, many questions still remain. It is still unclear if metformin will be effective in a non-diabetic population, and if its effects are limited to particular patient populations (e.g. specific malignancies or cancer cells with specific metabolic pathway mutations). Alongside this, much of the preclinical work has used supra-physiological doses of metformin. There are currently a large number of studies worldwide being undertaken examining metformin in a range of cancers; however, many of these are using metformin at the conventional anti-diabetic doses. It awaits to be seen if these doses will be effective or if further phase I and phase II clinical trials will be required to assess metformin’s tolerability and efficacy as an anti-cancer agent at higher doses. Well-designed prospective controlled clinical trial outcomes will be important to provide more definitive answers regarding the efficacy of metformin in prevention and treatment of prostate cancer, and its role as a potential adjuvant therapy. However, data so far is very compelling for metformin’s role in the treatment of prostate cancer, particularly in view of its potential synergy with currently used treatments such as ADT and radiotherapy.
